# Verification of the chromosome number using cytogenetics and estimation of genome size via flow cytometry and *k*-mer analyses for representative *Anoectochilus roxburghii* accessions

**DOI:** 10.1371/journal.pone.0322457

**Published:** 2025-05-28

**Authors:** Wenting Zhang, Yu Mei, Jihua Wang

**Affiliations:** Crop Research Institute, Guangdong Academy of Agriculture Sciences/Guangdong Provincial Key Laboratory of Crops Genetics and Improvement Guangdong/Guangdong Provincial Engineering & Technology Research Center for Conservation and Utilization of the Genuine Southern Medicinal Resources, Guangzhou, Guangdong, China; Government Degree College Totakan, PAKISTAN

## Abstract

*Anoectochilus roxburghii* (Wall.) Lindl. is a perennial herb of orchidaceae. Because of its functions of heat-clearing and blood-cooling, removing dampness, detoxification and enhancing immunity, it is considered as a nutritious medicinal plant with high economic value. Originating from Guangdong province, China, chromosome number and genome size of *A. roxburghii* (“Luofushan-1”) were determined through cytogenetics, flow cytometry, and k-mer analysis. We analyzed the karyotype of *A. roxburghii* using different cytogenetic markers, and the results showed a chromosome number of 2n = 80, but no sex-linked chromosome heterotropy was observed. For flow cytometry analysis, tomato and maize were used as internal standard, and the 2C DNA amount among sixteen Anoectochilus accessions ranged from 6.57 to 8.26 pg (including Luofushan-1). Additionally, a genome survey of “Luofushan-1” was performed using Illumina HiSeq 2000 DNA sequencing, k-mer analysis revealed that the genome size of “Luofushan-1” was approximately 5.68 Gbp. This comprehensive study establishes a foundation for subsequent whole-genome sequencing of A. roxburghii, contributing valuable insights into its genetic characteristics and paving the way for further research on this medicinal plant.

## Introduction

Species within the genus *Anoectochilus*, belonging to the Orchidaceae family, are rare perennial herbs primarily found in Oceania, Asia, tropical and subtropical areas, as well as in Zhejiang, Fujian, Taiwan, Guangdong, Yunnan and Guangxi, spanning from the southern to the eastern and western parts of China (Table 1). Recognized by its distinctive golden venation throughout its leaves, this genus is commonly referred to as “Jinxianlian” in Chinese.

**Table 1:** The principal species within the genus Anoectochilus.

Species name	Origin/Main distribution	Reference
*Anoectochilus acalcaratus*	Vietnam	[62, 63]
*Anoectochilus annamensis*	Vietnam	[64]
*Anoectochilus brevilabris*	Yunnan, China	[65]
*Anoectochilus burmannicus*	Thailand	[66, 67]
*Anoectochilus calcareus*	Guangxi, China	[68]
*Anoectochilus calcareus*	Vietnam	[69]
*Anoectochilus elatus*	India	[70]
*Anoectochilus elwesii*	Yunnan, China	[71]
*Anoectochilus emeiensis*	Sichuan, China	[72]
*Anoectochilus formosanus*	Taiwan, China	[73]
*Anoectochilus koshunensis*	Taiwan, China	[74, 75]
*Anoectochilus longilobus*	Yunnan, China	[76]
*Anoectochilus lylei*	Yunnan, China	[77]
*Anoectochilus malipoensis*	Yunnan, China	[63]
*Anoectochilus nandanensis*	Guangxi, China	[68,78]
*Anoectochilus regalis*	India	[79]
*Anoectochilus roxburghii*	Fujian, China	[80]
*Anoectochilus sandvicensis*	USA	[81]
*Anoectochilus setaceus*	Vietnam	[82]
*Anoectochilus sikkimensis*	India	[79]
*Anoectochilus xingrenensis*	Guizhou, China	[83]
*Anoectochilus zhejiangensis*	Zhejiang, China	[84]

Among the *Anoectochilus* species, *Anoectochilus roxburghii* (Wall.) *Lindl*. and *Anoectochilus formosanus* stand out as the most extensively reported and researched. Renowned for their diverse applications in traditional medicine, they are often referred to as the “king medicine” and the “golden herb” [1].

*A. roxburghii* holds substantial economic potential in both local and export markets due to its properties such as heat dissipation, blood cooling, detoxification, dampness elimination, and immunity enhancement [2]. The main active components of *A. roxburghii*, including polysaccharides comprising glucose, arabinose, xylose, galactose, rhamnose, and mannose, exhibit antioxidant, hypoglycemic, and anti-tumor effects [3]. Thus, *A. roxburghii* has received more and more attention in the fields of health products, cosmetics, food and others.

*A. roxburghii* likes cool, wet, and especially prefers to grow under the evergreen broad-leaved trees of the ditch, stone walls, and loose soil in the wet zone. Due to the huge medical and economic properties, the natural resources of *A. roxburghii* have been highly exploited which makes the wild *A. roxburghii* over-excavated. In order to meet the market demand, the breakthrough has been made in the tissue culture propagation technology of *A. roxburghii* [4–6]. The large-scale planting of *A. roxburghii* lays the foundation for its industrial development [7]. While asexual propagation has been successful in meeting market needs, it has resulted in minimal genetic variation within or between populations of A. roxburghii. This limited genetic diversity poses challenges to breeding and selecting improved varieties for commercial purposes.

In the cultivation of A. roxburghii, various challenges persist, including the unregulated breeding of varieties, leading to a lack of a standardized variety breeding system. This has resulted in significant degeneration among different *A. roxburghii* varieties. Consequently, determining the genetic background and information of distinct Anoectochilus resources becomes crucial. A comprehensive investigation into the genetic relationships and evolutionary patterns among Anoectochilus resources in different regions is vital to address issues of resource confusion in production [8].

In addition, most of the current studies on Anoectochilus focus on pharmacological components and pharmacological activities, and few studies on genome size, chromosome analysis and ploidy level [9,10]. The exploration of nuclear genome size and chromosome numbers is pivotal for gaining a comprehensive understanding of the genetic aspects of A. roxburghii.

Analyzing the chromosome count in species with many chromosomes involves several technical, biological, and methodological challenges [11,12]. Therefore, advanced techniques and careful preparation are essential to obtain accurate results. And, achieving high-quality chromosome preparations is especially important for reliable chromosome count and karyotype analyses. Fluorescence in situ hybridization (FISH) stands out as a pivotal technique for localizing specific nucleic acid sequences within cells or tissues, allowing for the visualization of gene expression, chromosomal abnormalities, and the arrangement of DNA/RNA molecules in their natural context. Leveraging labeled molecular markers like repetitive elements or single-copy genes, FISH enables the determination of the genomic location of target DNA. This technique plays a crucial role in elucidating phylogenetic relationships, genome organization, polyploidy, and chromosome mapping in various species [13]. FISH allows the visualization of DNA/RNA associated with specific genomic regions or entire chromosomes across a spectrum of cytological specimens [14].

A genome encompasses all the information required for an individual’s development and functioning, comprising coding and non-coding DNA, mitochondrial DNA, and chloroplast DNA. The term “2C DNA amount” refers to the total DNA content in the haploid genome of a diploid organism, representing the amount of DNA in a non-replicating diploid nucleus [15]. The size of a species’ genome can typically be represented in two primary ways: by weight or by the number of base pairs. Picograms (pg) is a frequently employed unit when measured by weight. Alternatively, the genome size can be expressed through the count of base pairs, utilizing standard units such as kilobase (Kb), megabase (Mb), and gigabase (Gb). The representation using the number of base pairs is especially advantageous in the context of genome sequencing, as it provides a precise count of the nucleotide composition. Conversely, the weight-based representation is commonly utilized when discussing the amount of nuclear DNA content within a species, offering a macroscopic perspective on the genomic material. Genome size plays a crucial role in genome sequencing projects, as it determines the sequencing depth budget and offers an initial estimate for assessing the completeness of genome assembly [16].

In the realm of plant biology, flow cytometry (FCM) has emerged as the preferred method for determining ploidy and genome size due to its rapid, straightforward, and accurate nature [17–19]. FCM is a contemporary cell analysis technology that utilizes flow cytometry for multi-parameter, rapid quantitative analysis of particles in the fast linear flow state of single-cell organisms, concurrently attempting to sort them into specific groups [20].

Furthermore, with the rapid advancements in next-generation sequencing (NGS), genome size estimation can also be achieved through *k*-mer analysis of Illumina sequencing data [21,22]. NGS generates vast amounts of sequencing data, allowing for the comprehensive analysis of *k*-mer within these sequences. Using hashing algorithms, researchers can quickly count the occurrences of each *k*-mer in the sequencing data, providing insights into the sequence composition and structure [23,24]. NGS contributes to *k*-mer analysis by providing high-throughput sequencing data, which enables efficient *k*-mer counting, genome size estimation, assessment of sequencing quality, evaluation of genome heterozygosity and repeated sequences, detection of genetic variants, and various other bioinformatics applications.

In this investigation, FISH utilizing 5S and 18S rDNA probes, along with telomere repeat sequences from Arabidopsis, was employed to ascertain the chromosome number and karyotype of “Luofushan-1”. Simultaneously, the genome size of *A. roxburghii* was determined through flow cytometry and *k*-mer analysis. Accurate details regarding chromosome number and genome size are pivotal for providing essential genetic information crucial to the research and production endeavors related to A. roxburghii.

## Materials and methods

### Plant materials

**Fig 1:**
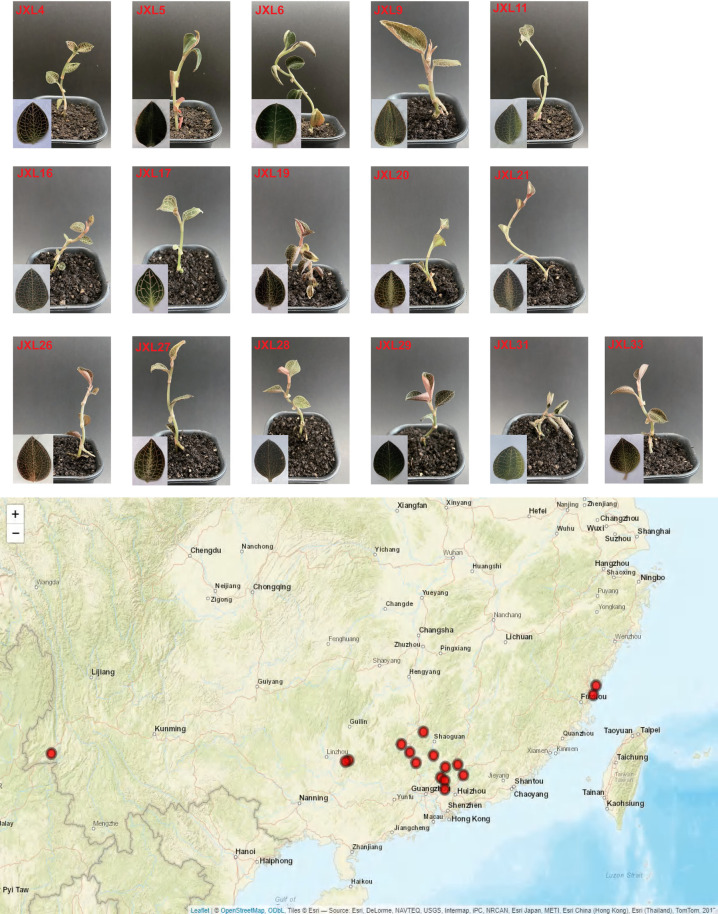
5–8-month-old Anoectochilus plants.

We performed stable tissue culture of *A. roxburghii* collected from several provinces in southern China (mainly Guangdong) among which JXL28 specifically refers to “Luofushan-1”, selected as a representative local cultivar from Luofu Mountain in Guangdong Province, exhibits exceptional traits such as robust heat resistance and rapid growth (Fig 1, Table 2). To make it simpler and clear, we use numbers instead of specific names when referring to these accessions in the main text and specify Anoectochilus roxburghii as their scientific name. For FCM analysis, fresh and young leaves were obtained from tissue-cultured plantlets of *A. roxburghii* that were approximately six months old and had attained a height of 6–7 centimeters. and utilized for the subsequent analysis. As for chromosome counting, young and whitish root tips (2–3 cm) were harvested from aerial roots of plants of JXL28.

**Table 2:** Plant materials employed in the study.

Accessions	Collection location
JXL4; JXL 5	Heyuan, Guangdong
JXL6; JXL26	Fujian
JXL9; JXL11	Guangxi
JXL16; JXL17; JXL28	Huizhou, Guangdong
JXL19; JXL20; JXL21	Qingyuan, Guangdong
JXL27	Yunnan
JXL29; JXL31	Meizhou, Guangdong
JXL33	Shaoguan, Guangdong

### In vitro propagation, seedling hardening, and transplantation

Based on previous reports, we conducted in vitro propagation of Anoectochilus roxburghii after improving the culture medium formula. Specifically, stem segments of A. roxburghii, approximately 2.0 cm in length and with aerial roots, were excised and inoculated into an improved medium based on Murashige and Skoog (MS) medium, supplemented with 30 g/L sucrose and 6 g/L agar powder, adjusted to a pH value of 5.8. In the culture room, the lids of the tissue culture bottles were opened for 24 hours. Subsequently, tissue-cultured seedlings with 3–5 leaves and a height of over 4 cm were selected, and the medium adhering to their roots was rinsed off with water before transplanting them into seedling pots. The seedlings were then cultivated under conditions of a temperature of (22±2) ℃, a relative humidity of approximately 80%, and a light intensity of 400 lx for one more month.

### Chromosome preparation

Fixation: A 0.5ml centrifuge tube with holes at the top was prepared and moistened with water before the experiment. The clipped root tips were placed in the tube on ice. The root tips were treated with 0.9–1 mpa nitrous oxide for 2h in the inflatable tank, and then fixed in the 90% pre-cooled acetic acid for 10min. The fixed root tips were then transferred into 70% ethanol solution and stored at ‐20 °C until use.

Enzymatic hydrolysis and Crushing: Chromosomal slides were prepared following the method of Schwarzacher [25]. The fixed root tips were rinsed three times for 10 min in water. The root tip meristematic cells was treated with enzyme mixture in an enzyme buffer (cellulase:pectinase 3:1) at 37 °C for 60–90 min. In order to ensure optimum removal of cell wall and allow good spreading of metaphase chromosomes, the time of digestion was optimized according to previous reports [26–28]. After that, the root tip was cleaned three times with 70% alcohol, and the root tip was crushed and shaken in the remaining alcohol with an anatomical needle. The cells were collected by centrifugation at 4000r/min and suspended in 90% acetic acid solution again for tablets pressing.

### Probe preparation

DNA probes were synthesized by nick translation. The labeling reactions consisted of l ug genomic *A. roxburghii* DNA, 5 μl of dNTP, 2 μl of Fluorescein-12-dUTP (green) or Texas Red-5-dUTP (red), 5 μl of 10xNT (reaction buffer), 5 μl of dithiothreitol (DTT), 2 μl (4 U/μl) of DNA polymerase I, 1.8 μl DNaseI (1.2:1000), 28.2 μl of double distilled water, in a final volume of 50 μl.

### Fluorescence in situ hybridization with oligonucleotide probes

A small amount of water was added to the fluorescence in situ hybridization box to maintain the humidity, and the probe solution was configured according to the number of slides. A synthetic terminal (TTTAGGG)6 sequence was used as telomere probe, while the labeled pTa794 and pTa71 plasmid DNA were used as probes for 5S rDNA and 18S rDNA, respectively. 8 μl 1×TE 2×SSC (pH7.0) solution and 0.25μl probe solutions, as well as probes labeled with green and red fluorescence colors, were added to each slide. The slides were covered by coverslips and heated at 80 ℃ for 5 min, then placed in a hybridization box and hybridized overnight at 37℃ to 42℃. The slides were cleaned 2–3 times with 2×SSC solution at room temperature, then blow-dried and added with 8μl DAPI. The chromosomal karyotypes were accurately analyzed by high resolution fluorescence microscope CCD (Charge Coupled Device).

### Nuclear extraction and measurement

In FCM analysis, it is crucial that the sample be a suspension of a single intact particle to prevent blockages in the flow chamber orifice. Optimal conditions involve using well-watered plants and fresh material before analysis, considering metabolite accumulation in senescent tissue and the impact of a higher proportion of endopolyploid nuclei. Thus, 20mg fresh leaves of *A. roxburghii* were placed in a plastic petri dish. A universally accepted nuclear isolation buffer was selected based on previous studies, which found that Galbraith’s separation buffer [29] had the best dissociation effect on many plant species, organs and tissues [30]. Galbraith’s buffer supplemented with RNaseA, propidium iodide (PI) [31] and reducing agents mercaptoethanol and polyvinylpyrrolidone (PVP-40) [32] were utilised. Then, nuclei suspensions were extracted from young leaves through manual chopping using a sharp scalpel. The suspension of released nuclei was filtered into a tube through 40 μm nylon mesh and incubated in the dark for 0.5~1 h at 4 °C with occasional shaking to allow optimum PI intercalation with the DNA strands.

### Cytograph measurement

Using tomato and corn as the internal reference, the suspension of *A. roxburghii* (JXL28, also known as “Luofushan-1”) sample and reference sample were mixed in an appropriate ratio. The BD FACScalibur flow cytometer was used to detect the stained cell nucleus suspension. The fluorescence intensity of propidium iodide was detected by 488 nm blue light excitation. Histograms were obtained over 1000 events. The quality of the analysis was evaluated by estimating the proportion of background fragments, the symmetry of the detection peak, and the distribution of fluorescence intensity, expressed as a coefficient variation (CV% was controlled within 5%).

Graphical analysis was performed using Modifit 3.0 software. The peak values corresponding to the G0/G1 phase of both the determination samples and internal standard were obtained and recorded. The DNA content of samples was calculated according to the following formula: Sample DNA content = Reference DNA content × (sample 2C mean peak position/reference 2C mean peak position). The estimation of DNA content was based on corn B73 (2C DNA content = 4.70 pg) [33] and tomato Heinz 1706 (2C DNA content = 1.69 pg) [34] as the internal reference standard (1 pg = 978 Mb). All experiments were conducted at least three times.

### Genomic size estimation by *k*-mer analysis

*A. roxburghii* genomic DNA was extracted by CTAB method, and the purity and concentration of DNAs were detected by NanoDrop One (Thermo Fisher Scientific) spectrophotometer and Qubit 3.0 Fluorescence Spectrometer (Life Technologies, Carlsbad, CA, USA). A paired-end library with an insert size of approximately 350 bp was constructed using the Nextera DNA Flex Library Prep Kit (Illumina, San Diego, CA, USA), following manufacturer instructions, and was sequenced using the Illumina NovaSeq 6000 platform. Quality control of raw sequence data was done using FastQC. The reads were filtered before assembly to ensure that a pair of paired-end reads had more than 90% of bases with quality ≥Q30. High-quality cleaned Illumina sequences were subjected to *k*-mer counting using JELLYFISH [35] with different *k*-mer size (17–51). *k*-mer depth distribution was counted and the peak value of the depth distribution was identified. The mean *k*-mer depth equals the peak value of the *k*-mer depth distribution as the depth of the *k*-mer coverage following a Poisson distribution. The *k*-mer count distribution from Jellyfish was used to produce a report and several informative plots describing the genome properties by GenomeScope 2.0 [36].

## Results

### Flow cytometry analysis

**Fig 2:**
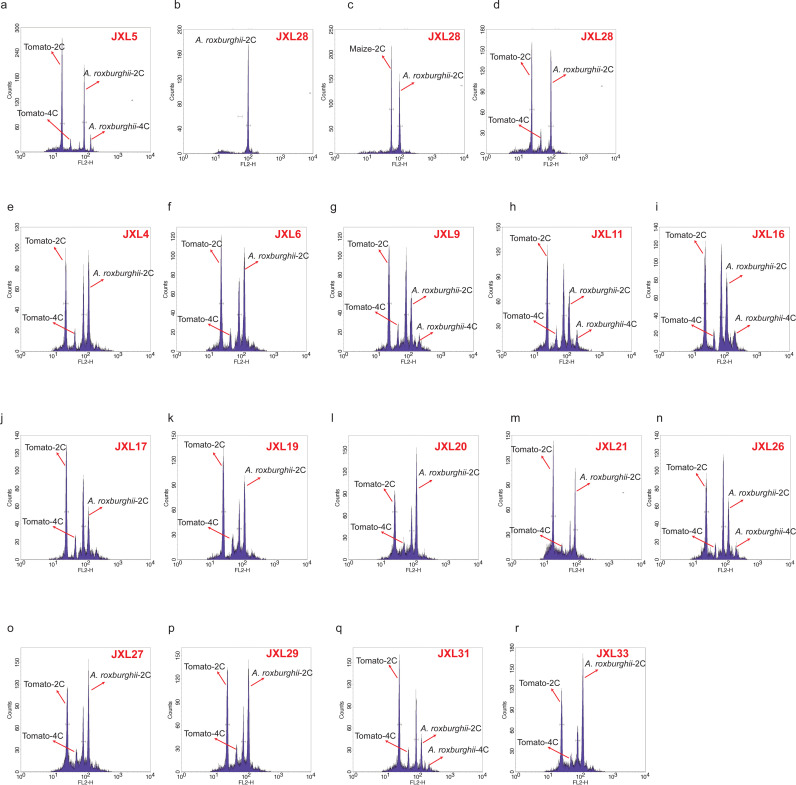
Histogram of fluorescence intensities in nuclei suspensions stained with PI isolated from leaves of 16 Anoectochilus species.

Healthy leaves from 16 *A. roxburghii* plantlets (Fig 1, Table 2), produced by in vitro culture were utilized for FCM analysis. The DNA 2C-value of *A. roxburghii* was determined by comparing the relative PI-fluorescence peak for nuclei of Solanum lycopersicum to that of *A. roxburghii* samples. The 2C peak of all these species did not overlap with the 2C peak of tomato. The fluorescence of nuclei isolated from leaves of these species were stable, and DNA histograms presented low background debris and relatively low CV values. The genome size of all 16 *A. roxburghii* accessions, estimated by flow cytometry, are presented in Table 3. Among them, JXL9 (2C =8.26pg, 8.08 Gb) had the largest genome size, while JXL28 (2C =6.57pg, 6.43 Gb) had the smallest. Even when the internal reference species was changed to maize, the estimated genome size of JXL28 still exhibited the smallest. Except for JXL28 and JXL5 (Fig 2a–d), most accessions exhibit a discernible pattern characterized by the presence of two prominent peaks (Fig 2e–r). Their cellular DNA fluorescence intensity peaked at ~82.04 and ~119.29, respectively, with a ratio of about 0.69. Upon proportional analysis, the fluorescence intensity of these two peaks does not align with each other, neither conforming to the expected ratio of 2C to 4C nor to the ratio of 3C to 4C. These data indicate that these accessions are mixoploids due to errors in chromosome replication or segregation and cells with two different chromosome numbers within the cell population. In conclusion, the nuclear DNA contents for *A. roxburghii* ranged from 6.57 to 8.26 pg per 2C-1.

**Table 3:** Flow cytometric estimation of genome size of 16 *A. roxburghii* species.

Sample	Reference	Average ratio of fluorescence intensity	2C-value (pg)	Total genome size (Gb)
JXL28	Maize	1.71±0.027	8.04±0.13	7.86
	Tomato	3.89±0.033	6.57±0.06	6.43
JXL5	Tomato	4.57±0.024	7.72±0.04	7.55
JXL4	Tomato	4.66±0.019	7.88±0.03	7.71
JXL6	Tomato	4.78±0.020	8.08±0.03	7.90
JXL9	Tomato	4.89±0.028	8.26±0.05	8.08
JXL11	Tomato	4.72±0.027	7.98±0.05	7.88
JXL16	Tomato	4.63±0.027	7.82±0.05	7.65
JXL17	Tomato	4.73±0.031	7.99±0.05	7.81
JXL19	Tomato	4.65±0.033	7.86±0.06	7.69
JXL20	Tomato	4.71±0.027	7.96±0.05	7.78
JXL21	Tomato	4.44±0.021	7.50±0.04	7.34
JXL26	Tomato	4.62±0.026	7.81±0.04	7.64
JXL27	Tomato	4.74±0.033	8.01±0.06	7.83
JXL29	Tomato	4.63±0.021	7.82±0.04	7.65
JXL31	Tomato	4.85±0.019	8.20±0.03	8.02
JXL33	Tomato	4.73±0.032	7.99±0.05	7.81

1 pg = 978 Mbp.

### Cytogenetic analysis

**Fig 3:**
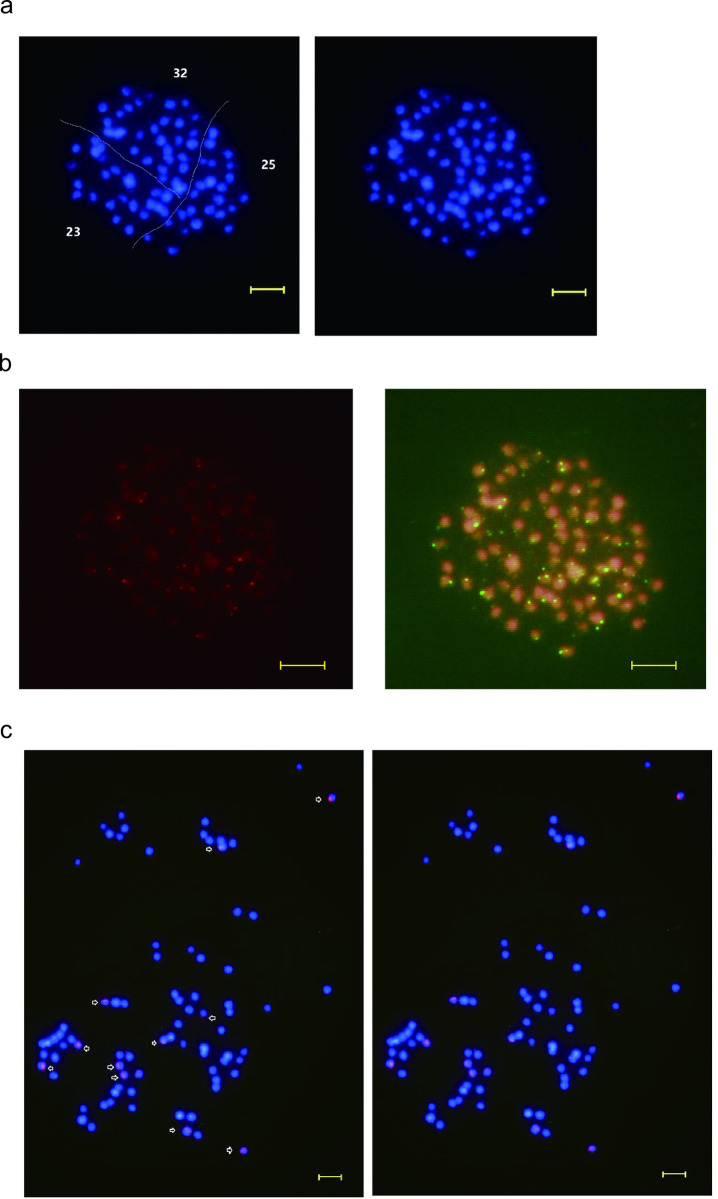
Somatic chromosomes analysis of *A. roxburghii* (JXL28). a, chromosome fluorescence staining results of JXL28 by DAPI; b, telomere fluorescence in situ hybridization results of JXL28; c, 5S rDNA probe fluorescence in situ hybridization results of JXL28; Scale bar = 5 μm.

To conduct a cytogenetic analysis and determine the chromosome number of JXL28, DAPI (4′,6-diamidino-2-phenylindole), a widely used fluorescent dye for nuclear staining, was employed. DAPI binds specifically to double-stranded DNA, effectively illuminating the nuclei of chemically fixed cells [37]. For this purpose, root tip cells of JXL28 were utilized for the preparation of chromosome samples, which were subsequently stained with DAPI fluorescent dye. Fig 3a displays detailed images from the chromosome count analysis, revealing an initial observation of 80 chromosomes in the JXL28 plant cells. Each of these chromosomes exhibited a range of lengths, varying from approximately 1.0 to 2.0 microns. This specific size range indicates small chromosomes, which can be challenging to distinguish and accurately count but were visible due to the efficient staining procedure and high-resolution imaging techniques employed. The uniformity and distinctiveness of these chromosomes provided valuable insights into the genomic structure and karyotype of JXL28, laying a foundational basis for further genetic and cytological studies.

Using FISH technology to pinpoint the rRNA gene (rDNA) or telomeric sequence on chromosome not only allows the determination of their positions but also provides effective markers for chromosome identification [26,38,39]. In most higher plants, chromosomes are characterized by the presence of Arabidopsis-type telomere sequences at their termini [40,41]. Thus, FISH mapping was performed to detect rDNA and telomeric repeats in the JXL28, and the results are depicted in Fig 3b. The green fluorescence signal represents the hybridization site of Arabidopsis-type telomeric repeats, demonstrating clear and stable signals localized to the chromosome termini (Fig 3b). The FISH results of the 5S and 18S rDNA probes to the mitotic chromosomes, previously stained with DAPI, are presented in Fig 3c. There were 12 hybridization sites with strong 5Sr DNA (red) on the JXL28 chromosome, but no significant hybridization signal of 18S rDNA was detected. The results also confirmed the polyploid nature of the JXL28 sample (2n=80). This study marks the first cytological characterization of both telomeres and rDNAs in A. roxburghii.

It is estimated that 5% to 10% of flowering plants exhibit genetic sex determination, suggesting around 30,000 species across various angiosperm taxa possess sex-linked genomic regions [42], some with heteromorphic sex chromosomes, while others have undetectable regions due to small size or lack of pairing/crossovers [43]. Similar to animal sex chromosomes, the sex-linked regions in plants exhibit signs of repetitive sequence accumulation and genetic degeneration. As an endangered polyploid plant, we are curious whether sex-linked genome regions can be detected in *A. roxburghii* through cytological analysis to explain the accumulation of repetitive sequences and genetic degeneration. Here, no sex-linked chromosome heterotropy in *A. roxburghii* was observed possibly due to small size of each chromosome.

### *k*-mer analysis predicts genome size

The paired-end 300 bp insert size library underwent sequencing on the NovaSeq Illumina platforms, generating 1.86 billion read pairs. Following adapter removal and quality filtering, the clean data exhibited an average read length of 143 bp. Considering the flow cytometry estimates of the JXL28 genome size (6.43 Gbp), the total genome coverage from the quality-processed data reached 41x. Initial steps involved calculating the *k*-mer distribution frequency using jellyfish, followed by genome size, duplication, and heterozygosity estimation of JXL28 using GenomeScope 2.0, a tool optimized for polyploid genomes.

Our analyses revealed that GenomeScope performance was notably influenced by parameter settings, leading to variable estimates of the total DNA amount in the haploid genome of JXL28, ranging from 0.719 Gbp to 2.0 Gbp (Table 4). The *k*-mer size emerged as the most influential parameter, with a gradual increase in genome size as the *k*-mer size increased. This trend suggests that larger *k*-mer sizes incorporate more genomic information, resulting in higher estimated genome sizes.

**Table 4:** Genome size estimates for JXL28 genotype (in bp) using quality processed Illumina sequencing data.

CovMax	k17	k19	k21	k25	k31	k41	k51	Average
1k	719,259,591	1,056,316,327	1,295,452,490	1,147,796,575	1,356,675,620	1,621,135,279	1,719,700,889	1,273,762,396
5k	910,837,485	1,318,405,726	1,137,476,562	1,580,156,348	1,569,322,077	1,823,351,041	1,866,019,423	1,457,938,380
10k	1,040,170,971	1,379,146,944	1,228,886,069	1,478,263,586	1,660,352,277	1,890,656,542	2,000,787,332	1,525,466,246
100k	1,040,170,971	1,379,146,944	1,228,886,069	1,478,263,586	1,660,352,277	1,890,656,542	2,000,787,332	1,525,466,246
Average	890,089,349	1,251,289,666	1,220,605,040	1,402,072,170	1,528,783,325	1,778,380,954	1,862,169,215	**1,419,055,674**

K17–K51 = k-mer sizes; CovMax = cutoff threshold for maximum k-mer coverage (varied for GenomeScope from 1k to 100k).

**Fig 4:**
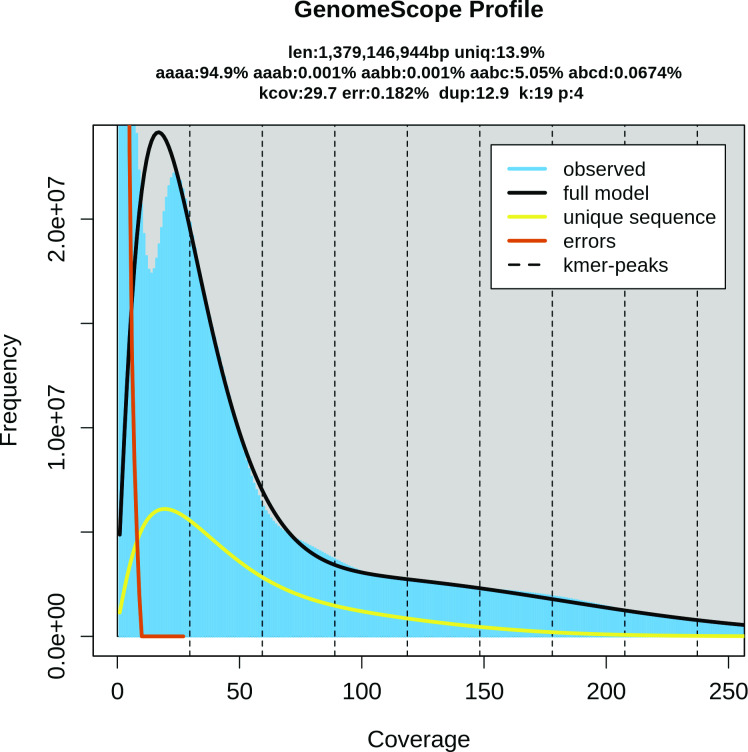
The 19 k-mer distribution of *A. roxburghii* (JXL28) as revealed by GenomeScope results.

The estimates of genome size increased with a higher cutoff threshold for maximum *k*-mer coverage (CovMax). Elevating the threshold from 1k (the default setting of GenomeScope) to 5k or 10k led to average increases in genome size estimates by 0.157 Gbp and 0.223 Gbp, respectively. Using higher thresholds (up to 100k), there were no substantial differences in genome size estimates among the investigated programs and parameter settings, yielding an average polyploid genome size of 1.52 ± 0.35 Gbp. Lower CovMax thresholds excluded highly covered *k*-mer, mainly associated with repetitive sequences, potentially causing a significant reduction in genome size estimates [16]. Based on *k*-mer analysis, the average polyploid genome size of JXL28 was estimated at 5.68 (1.42 plus 4) Gbp and the 19 *K*-mer distribution revealed by GenomeScope showed in Fig 4.

## Discussion

### Chromosome counts

Chromosome count analysis in *A. roxburghii* poses challenges due to the abundance of small chromosomes, and there have been limited studies on chromosome identification and karyotype analysis for this species. In Chinese journals, Zeng et al. reported a chromosome count of 2n=40 for *A. roxburghii* [44], while Xie et al. [8] identified two karyotype types, 2n=40 and 2n=80, through the analysis of nine different accessions. They suggested that the variation in karyotypes might be a key factor influencing the diverse plant architecture observed in A. roxburghii, proposing a correlation between chromosome count and plant size. Here is the first English report on karyotyping of A. roxburghii. In our study, we determined that the local accession “Luofushan-1” from Guangdong possesses 2n=80 chromosomes.

Research shows that within-population variability in herbivory increases with latitude, decreases with plant size implying that larger plant size may confer some protection against herbivory. Interestingly, when compared to the representative accession from Fujian with 2n=40, “Luofushan-1” did not exhibit a clear advantage in terms of plant size. Previous research has highlighted the significance of polyploidy in the adaptive evolution of plants [45]. Such as, the high frequency of polyploidy observed in the Arctic prompts us to delve deeper into the role that polyploidy plays in plants’ adaptation to extreme environments [46]. Moreover, plants regenerated from tobacco cells that were adapted to 428 mM NaCl were found to have hexaploid or near-hexaploid chromosome numbers compared to the normal tetraploid, suggesting that the hexaploid condition may impart some karyotypic stability that allows more efficient morphogenic activity [47]. Therefore, we speculate that the “Luofushan-1” accession, with 2n=80 chromosomes, may harbor specific adaptations that confer advantages in challenging or extreme environments. This suggests a nuanced and context-dependent relationship between chromosome count, plant architecture, and adaptation.

Besides, sex-linked genome regions are crucial for the sexual reproduction and genetic health of dioecious and polyploid plants. The accumulation of repetitive sequences and genetic degeneration in sex-linked regions may have implications for the genetic diversity of a population. These regions, often associated with sex chromosomes, may experience slower rates of recombination and higher rates of mutation. This can lead to a loss of genetic variation, potentially reducing the plant’s ability to adapt to changing environmental conditions or resist diseases [48]. Our results show that the chromosomes of *A. roxburghii* are relatively small, which may pose challenges for observing cytological features. In future studies, more advanced methods could be employed for more detailed observations. The ability to detect sex-linked genome regions through cytological analysis can be valuable for understanding the underlying genetics of sex determination and reproductive strategies in endangered species. Identifying sex-linked markers or regions susceptible to genetic degeneration can provide insights into the plant’s evolutionary history and help guide conservation efforts [48,49].

Telomeres represent unique chromosomal structures composed of tandem repeats situated at the ends of chromosomes, intricately associated with sheltering proteins [50]. Functionally, telomeres play a crucial role in preserving chromosome integrity and individuality. These sequences typically consist of tandemly arranged minisatellites, often following the pattern (TxAyGz)n, with the prevalent plant-type telomere sequence being TTTAGGG, originally identified in Arabidopsis thaliana [51]. In our investigation, we utilized the telomere sequence of the Arabidopsis type. The hybridization signal was localized to the terminal regions of *A. roxburghii* chromosomes in proximity to the telomere region, exhibiting some variations in signal strength. This discrepancy could be attributed to the chromosome specimen preparation process, where parts of the chromosomes might not be fully extended, resulting in weaker or absent signals. Additionally, signal variations might arise due to potential overlaps between telomeric and silk grain regions, contributing to signal enhancement.

### Chromosomal localization of 5S rDNA loci

Eukaryotic rDNA encompasses genes that encode various rRNA precursors, including 5S rDNA and 45S rDNA, which are highly conserved tandem repeat sequences. The chromosomal locations of these sequences vary across species [52]. 5S rDNA and 45S rDNA serve as robust cytogenetic markers. The 5S rDNA gene family typically exists in the genome as high-copy tandem repeats and is a constituent of the large subunits of most biological ribosomes. The coding region of 5S rDNA is more conservative than the spacer sequences, with substantial differences in spacer sequences. Notably, the sequence in the middle of the spacer region exhibits the most abundant changes [53].

On the other hand, 45S rDNA is highly transcribed and expressed during the interphase of cell division, forming specialized regions involved in nucleolus formation. Within the 45S rDNA, 18S rDNA constitutes a portion, and its loci and localization function are expected to align with 45S rDNA [54]. However, in this study, only 5S rDNA hybridization signal sites were observed in *A. roxburghii* chromosomes, and no apparent 18S rDNA hybridization signal was detected.

### Nuclear DNA content analysis

Flow cytometry stands as a single-cell technology, gauging scatter and fluorescence to discern unique cellular properties [55]. It has become the primary method for measuring nuclear DNA content in plants, utilizing DNA fluorescent dye due to its ease of sample preparation and ability to conduct high-throughput measurements [56]. FCM is generally more advantageous than alternative methods, such as Feulgen densitometry, for estimating genome size, polyploid generation level, nuclear replication status, and endopolyploidy (polysomy) [30].

Propidium iodide (PI) fluorescent dye was employed for staining, as it can intercalate into double-stranded DNA without base-dependent bias, making it suitable for estimating DNA content in absolute units, provided RNA is removed with RNase [30]. In determining internal reference standards, it is advisable to select plants with known genome sizes, ensuring the internal standard is close to the genome size of the sample being tested, albeit not too close to avoid overlapping fluorescence peaks. Corn and tomato were used as internal standards in this study to measure the genome size of A. roxburghii, and the results demonstrated non-overlapping fluorescence peaks with good differentiation. The main reason for different standards leading to differences in the perceived size of nuclear DNA in flow cytometry is that these standards may contain distinct chemical components. These components can potentially interfere with or alter the binding efficiency of fluorescent dyes to DNA.

While genome size is generally considered constant at the species level, intraspecific differences were observed and characterized in various plant species [57]. Discrepancies between our results (2C-value: 6.57 to 8.26 pg) and previous findings (2C-value: 6.83±0.067 pg) [58], aside from differences in instruments and measurement techniques, are likely attributed to intraspecific variations. As we previously reported, the morphological traits of *A. roxburghii* differ significantly among accessions [59]. Various factors, such as genetic variation, environmental adaptability, and the complexity of genome structure may cause this intraspecific variation.

### Estimation of genome sizes by *k*-mer analysis

Utilizing whole genome sequencing, *k*-mer analysis emerges as a potent tool for estimating genome size by statistically analyzing the distribution of *k*-mer within sequencing fragments. This method has proven successful in estimating the genome size of species lacking pertinent genomic information, offering innovative insights into WGS and sequence assembly. Based on *k*-mer analysis, the genomic size of *A. roxburghii* (Luofushan-1) is estimated to be 5.68 Gb (1.42 multiplied by 4). This value slightly deviated from estimates obtained through flow cytometry, indicating potential variability between the two methods.

Upon comparison, GenomeScope stands out as an exceptional program. However, it’s noteworthy that genome size estimates are notably influenced by parameter settings, particularly *k*-mer size and coverage thresholds (CovMax). The estimated genome size using *k*-mer analysis is approximately 0.75 Gb below the value obtained through flow cytometry. Similar disparities have been observed in other studies, such as those involving the model plant Arabidopsis thaliana [60] and European eel [61]. These differences are thought to be associated with potential interference from chemical compounds affecting the stoichiometric measurements of DNA content in flow cytometry analyses. The exact genome size requires confirmation by additional genome-sequencing.

## Conclusion

The genomic size of an organism is a crucial metric, and in this study, a comprehensive approach involving cytogenetics, flow cytometry, and *k*-mer analysis was employed to determine the chromosome number and genome size of a representative accession of *A. roxburghii* from Guangdong. Notably, the results obtained from FCM and *k*-mer analysis are closely aligned. This study marks the pioneering report on the genome size of *A. roxburghii* using both FCM and *k*-mer analyses. The findings indicate that *A. roxburghii* possesses 2N=80 chromosomes, and its estimated genome size is approximately 5.68 Gb. This outcome not only lays the groundwork for subsequent whole-genome sequencing endeavors concerning *A. roxburghii* but also furnishes essential information for further genomic investigations of this economically significant species.

## Abbreviations

DAPI: 4′,6-diamidino-2-phenylindole
FISH: fluorescence in situ hybridization
FCM: flow cytometry
PI: propidium iodide
PVP: polyvinylpyrrolidone
WGS: whole genome sequencing
